# Harnessing the potential of shared data in a secure, inclusive, and resilient manner via multi-key homomorphic encryption

**DOI:** 10.1038/s41598-024-63393-1

**Published:** 2024-06-13

**Authors:** David Ha Eun Kang, Duhyeong Kim, Yongsoo Song, Dongwon Lee, Hyesun Kwak, Brian W. Anthony

**Affiliations:** 1 Department of Mechanical Engineering, Massachusetts Institute of Technology, Cambridge, USA; 2 Intel Labs, Hillsboro, USA; 3 Department of Computer Science and Engineering, Seoul National University, Seoul, South Korea

**Keywords:** Statistical learning, Data privacy, Multi-key homomorphic encryption, Multi-party collaborative learning, Dynamic membership, Adaptive machine learning systems, Computer science, Statistics

## Abstract

In this manuscript, we develop a multi-party framework tailored for multiple data contributors seeking machine learning insights from combined data sources. Grounded in statistical learning principles, we introduce the Multi-Key Homomorphic Encryption Logistic Regression (MK-HELR) algorithm, designed to execute logistic regression on encrypted multi-party data. Given that models built on aggregated datasets often demonstrate superior generalization capabilities, our approach offers data contributors the collective strength of shared data while ensuring their original data remains private due to encryption. Apart from facilitating logistic regression on combined encrypted data from diverse sources, this algorithm creates a collaborative learning environment with dynamic membership. Notably, it can seamlessly incorporate new participants during the learning process, addressing the key limitation of prior methods that demanded a predetermined number of contributors to be set before the learning process begins. This flexibility is crucial in real-world scenarios, accommodating varying data contribution timelines and unanticipated fluctuations in participant numbers, due to additions and departures. Using the AI4I public predictive maintenance dataset, we demonstrate the MK-HELR algorithm, setting the stage for further research in secure, dynamic, and collaborative multi-party learning scenarios.

## Introduction

### Benefit of learning from large pool of data

In machine learning, learning from a large pool of data is generally better than from a small dataset^1^. The theory of statistical learning illustrates this with bounds on the Rademacher complexity, a metric that assesses a model’s ability to generalize to new data. This bound shows a $1/\sqrt{n}$ relationship with data size of $n$, indicating that bigger datasets are more likely to represent the true underlying distribution^2^.

### Data aggregation challenge: data privacy

Amassing a large dataset often involves pooling data from multiple contributors^1^. However, this process presents the significant challenge of data privacy^3^. When these contributors are individuals, safeguarding their data privacy is unquestionably vital^4^. On the other hand, when the contributors are companies, the fact that their datasets can be combined implies they collect similar data, hinting at direct competition. As a result, these companies, potentially competitors, have substantial reservations about sharing their data, emphasizing the critical nature of addressing privacy risks when considering data aggregation^3^.

### State-of-the-art approach 1: federated learning

Federated Learning (FL) is an architectural strategy that enables a model to learn from individual participants’ data without compromising their privacy. This distributed learning approach trains algorithms across multiple decentralized edge devices. The basic idea is to share gradients instead of original data, thereby protecting each participant’s data privacy^5^. While FL has demonstrated its applicability in various industrial settings such as medicine^6^, resource optimization^7^ and prognostics and health management^8^, FL is prone to attacks like membership inference and gradient inversion, as an attacker can construct attack models that distinguishes members from non-members^9^, and gradients still carry critical information about input data^10^.

### State-of-the-art approach 2: homomorphic encryption

In this study, we focus on an alternative family of methods that leverages an encryption scheme to secure each member’s data, encouraging them to share it to a central server that exclusively performs the learning task. This approach, which assigns the entire computational load to the server, eliminates the need for intricate communication between participants, making it more feasible in situations involving a multitude of participants who have limited cryptographic communication capacities. This becomes achievable through Homomorphic Encryption (HE)^11^, a powerful encryption technique that enables the server to compute directly on the clients' encrypted data. To facilitate this, members generate a common public key, which is subsequently used to encrypt their data, following a multi-party protocol known as the threshold variant^12,13^.

### Limitation of state-of-the-art: practicality

The key limitation in the threshold variant is its requirement to fix the number of participants before the learning process begins^14^. This constraint is problematic for several reasons. First, not all contributors can join simultaneously due to the logistical challenges of collecting data in complex industrial environments. For some entities, data may become only available at a later point in time, making them available to join in the next round of learning. Second, it precludes late adopters from both contributing their data and benefiting from the evolved model. They not only miss out on the opportunity to share their unique data sets but also on the potential improvements made to the model. Third, the system’s inability to cope with member dropouts—a common feature in real-world settings—poses a threat to the continuity and robustness of the model. If the participant count must remain constant and a member leaves, the entire learning process would come to a halt, disrupting the collaborative benefit for the remaining participants.

These critical drawbacks reinforce the need for a more flexible, inclusive, and enduring approach to learning from aggregated data. Consequently, we need models that are capable of continuously onboarding fresh data sources and seamlessly adapting to participant transitions, all while ensuring an uninterrupted learning experience for everyone involved.

### Our contribution

In this study, we extend our previous work on single-party^15^ by introducing the MK-HELR (Multi-Key Homomorphic Encryption Logistic Regression) algorithm. This algorithm, tailored for a multi-party context, is founded on the multi-key HE construction^16,17^. It is designed to integrate new member data at any stage of the logistic regression learning process. Remarkably, it requires no prior action from existing members and guarantees data privacy for all participants. We believe our algorithm is especially pertinent to real-world scenarios as it adjusts to variable data availability, fosters inclusivity for late adopters, and remains resilient against participant dropout. To illustrate the potential applications of our algorithm, we demonstrate its usage through a publicly available predictive maintenance dataset (AI4I) from the UCI Machine Learning Repository^18^. Lastly, we briefly discuss the potential of our method within some industrial frameworks that entail a blend of collaboration and competition. We envision our study to lay the foundation for future research into dynamic, inclusive learning algorithms that capitalize on the benefits of learning from aggregate data.

## Motivation

### Theoretical motivation

In this section, we briefly illustrate the theoretical underpinnings of why learning from larger datasets is likely to enhance statistical machine learning outcome.

#### Excess risk

Excess risk is a metric that evaluates the generalization capability of a machine learning model. If we define $L$ as the expected risk, given by the formula ${\mathbb{E}}_{\left(x,y\right)\sim P}\left\{l(y, f\left(x\right)\right\}$ where $x$ and $y$ are inputs drawn from distribution $P$, $f$ is our model, and $l$ is the loss function, the excess risk can then be represented as in Eq. (1). Here, $w$ is the weight vector, and $\widehat{{w}_{\lambda }}$ is the solution that minimizes the regularized empirical risk.

1 $$L\left( {\widehat{{w_{\lambda } }}} \right) - inf_{{w \in R^{d} }} L\left( w \right)$$

This metric represents the difference between the expected prediction error of a learned model and the minimum error achievable by an ideal model that captures the perfect relationship between input features and target labels. Simply put, it measures how much worse the learned model is compared to an ideal model with complete knowledge of the true underlying relationship between the input and the target.

#### Excess risk decomposition

Excess risk can be decomposed into two components: the approximation error and the estimation error. The approximation error is a deterministic term that accounts for the model’s structural limitation, which can be reduced by using more complex models. For a given model, it is the estimation error that we seek to minimize during training. Estimation error occurs due to the finite size of the training dataset, because the sample of training data is only a finite representation of the true population.

Given a loss function $l:{\mathbb{R}}\to {\mathbb{R}}$, if there exists a constant ${C}_{0}>0$ such that $l(0)\le {C}_{0}$, then the estimation error $\mathcal{V}\left(\lambda \right)$ can be formulated as in Eq. (2). In this expression, $\widehat{L}$ represents the empirical risk and is defined as $\widehat{L}\left(f\right)=\frac{1}{n}\sum_{i=1}^{n}l({y}_{i}, f({x}_{i}))$, with $B=\sqrt{\frac{{C}_{0}}{\lambda }}$.

2 $${\text{V}}\left( \lambda \right) = 2 sup_{\left| w \right| \le B} \left| {L\left( w \right) - \hat{L}\left( w \right)} \right|$$

#### Performance Metric: Rademacher Complexity

Using the Jensen inequality, it is known that Eq. (3) holds:

3 $${\text{V}}\left( \lambda \right) \le 2Rad\left( {{\text{H}}_{B} } \right)$$

Here, $Rad\left({\mathcal{H}}_{B}\right)$ represents the Rademacher complexity of the ball ${\mathcal{H}}_{B}=\{w\in {R}^{d} | \Vert w\Vert \le B\}$. This is defined as ${\mathbb{E}}_{\text{\rm Z},\upvarepsilon } {sup}_{\left|w\right|\le B}|\frac{1}{n}\sum_{i=1}^{n}{\varepsilon }_{i}l({w}^{T}{x}_{i}{\text{y}}_{\text{i}})|$, which is the expected supremum of a linear combination of random variables. The coefficients (${\varepsilon }_{i}$) in this combination are random signs, referred to as Rademacher variables, with $P\left({\varepsilon }_{i}=\pm 1\right)=\frac{1}{2}$. In simpler terms, the Rademacher complexity quantifies a model’s ability to fit random fluctuations in the training data. A higher Rademacher complexity suggests that our model may overfit the training data, leading to poor generalization. Hence, controlling the upper bound of the Rademacher complexity becomes important.

Fortunately, for a loss function $l$ that is convex and Lipschitz, the contraction inequality ensures that:

4 $${\text{Rad}}({\text{H}}_{B} ) \le \frac{{2C_{L} B\kappa M}}{\sqrt n }\sim \frac{1}{\sqrt n }$$

Here, $\kappa$ and $M$ are positive constants satisfying $\Vert X\Vert \le \kappa$ and $\Vert Y\Vert \le M$, and ${C}_{L}$ is the Lipschitz constant. As shown, the upper bound exhibits a $1/\sqrt{n}$ dependence on the data size $n$. This provides insight into why larger datasets, which are likely to include more representative samples from the underlying distribution, can potentially yield higher quality learning outcomes.

#### Why logistic regression?

In our study, we employ logistic regression primarily to ensure our empirical analysis aligns with the theoretical foundations outlined in “Theoretical Motivation” Specifically, two aspects of logistic regression ensure this alignment. First, logistic regression fulfills a key theoretical assumption of regularized empirical risk minimization with margin loss functions, which requires the loss function to be both convex and Lipschitz. Second, in the context of linear models like logistic regression, the infimum ${inf}_{w\in {R}^{d}}L(w)$ used in defining excess risk is always attainable. This means an optimal ${w}^{*}\in {R}^{d}$ exists for which ${inf}_{w\in {R}^{d}}L\left(w\right)=L({w}^{*})$. Notably, this property holds true for functions within any finite-dimensional Hilbert space.

### Empirical motivation

For empirical exercise, we take a predictive maintenance dataset from the UCI Machine Learning Repository^18^, consisting of 10,000 data points. Each entry has 6 features, including air temperature, process temperature, and rotational speed, and carries a binary label where “1” signifies machine failure. For predictions, we use logistic regression as given by $\widehat{y}=\sigma ({w}^{T}x)$, where $x$ encompasses the features, $w$ is the weight vector, and $\sigma$ represents the sigmoid function $\sigma \left(z\right)=\frac{1}{1+{e}^{-z}}$. Notably, positive labels account for only 3.4% of the entries, marking an imbalanced dataset that closely mirrors real-world manufacturing scenarios.

**Figure 1:**
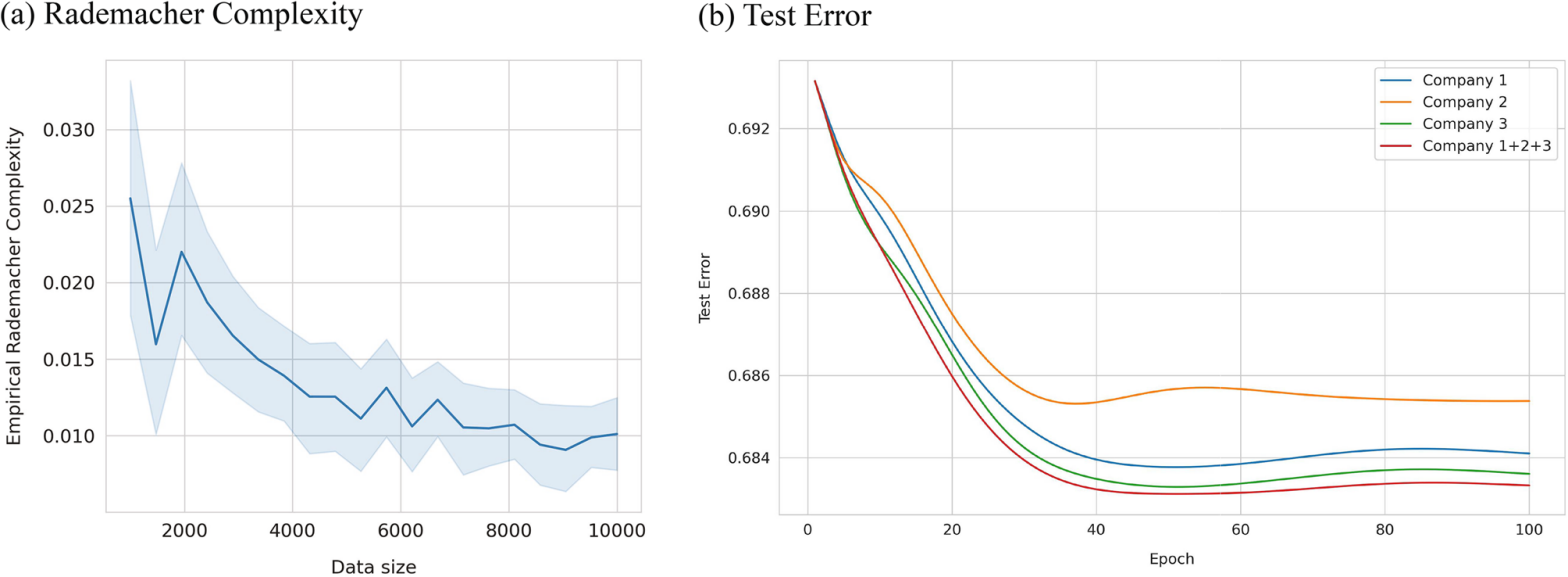
Benefit of data aggregation. (**a**) The empirical Rademacher complexity is plotted against data size, highlighting a decreasing trend that suggests data aggregation enhances model generalizability. (**b**) Test error in relation to the number of epochs for three separate entities and a combined unit is plotted, illustrating that data aggregation can diminish test error.

To empirically illustrate the advantages of data aggregation, we use this dataset to observe if the empirical Rademacher complexity diminishes with increasing data size ($n$). As demonstrated in Fig. 1a, the complexity indeed displays a downward trend, signifying that data aggregation mitigates overfitting and enhances model generalizability. For this analysis, the epsilon vector ($\varepsilon$) of size $n$ was drawn 50 times, with each entry being either + 1 or −1 with equal likelihood. The weight vector ($w$) was similarly drawn from a unit sphere 50 times to compute the empirical complexity.

Subsequently, we examined if whether data aggregation leads to reduced test error. Test error is a critical measure in statistical learning, often used to assess a model's performance. It quantifies the discrepancy between the model’s predictions and the actual labels on a separate test dataset, thereby evaluating the model's ability to generalize from the training data to unseen data. In the context of our study, the test error is computed as the negative log-likelihood, defined as $\sum_{i=1}^{n}-{y}_{i}\text{log}\left({\widehat{y}}_{i}\right)-\left(1-{y}_{i}\right)\text{log}(1-{\widehat{y}}_{i})$, where ${y}_{i}$ is the true label being 0 or 1, and ${\widehat{y}}_{i}$ is the predicted value for the $i$th data point.

Using a consistent random seed of 42 for reproducibility, we first partitioned a separate test dataset by randomly sampling 20% from the original data. The remaining data was then randomly divided into three subsets, assuming that each subset comes from a unique entity or company. As depicted in Fig. 1b, in our dataset, models trained on aggregated data demonstrated lower test errors compared to those trained on individual datasets.

These results not only empirically validate the theoretical merits of data sharing we previously addressed but also underscore the need to design a privacy-preserving tool optimized for learning from aggregated data. It is imperative to note that these plots don’t imply a guaranteed improvement in model performance. Instead, they serve as first-order empirical tests, with outcomes consistent with theoretical underpinnings. This motivates the development of a privacy-preserving toolkit tailored for statistical learning on data contributed by diverse sources.

Despite the potential advantages, sharing data—especially with competitors—raises significant data privacy concerns. In any competitive industry, safeguarding proprietary data is crucial. Specific patterns in variables like rotational speeds, temperature changes, or unique machine settings can reveal innovative production methods or exclusive manufacturing processes that give a firm its competitive advantage. Unintended disclosures could lead to financial losses, diminished market share, and a tarnished reputation. Therefore, it is essential to develop a tool that ensures data privacy throughout the data aggregation and machine learning process.

## Methods

### Homomorphic encryption

Homomorphic Encryption is an encryption technique that obviates the need for participants to possess cryptographic knowledge during the computation. This is achieved by enabling the server to handle the computation entirely on its own. In essence, all that participants do is encrypt their data and transmit it to the server. Subsequently, the server computes directly on the clients’ encrypted data. Upon decryption, the result is almost identical to that obtained if the operation had been performed on the raw, unencrypted data.

In a single-party setting, data can be protected using just one secret key ($s$) since only one party is involved. More specifically, the data owner can generate a ciphertext $\left(a, b\right)$ such that only the secret key owner can decipher it back to the original data ($m$) by carrying out a dot product between the ciphertext and the secret key, as shown in Eq. (5). For a detailed explanation of this process, refer to Supplementary Information S1. We omit this part from the main text, given that it is a well-known encryption technique, not our original contribution.

5 $$\left( {a, b} \right) \cdot \left( {1, s} \right) \approx m$$

However, in a multi-party context where the parties may not fully trust each other, a different approach is required.

### Prior art in multi-party settings

#### Threshold variant approach

In multi-party settings, a common method is the threshold variant approach^13,19^. This method asks data contributors to work together to create a shared public key to encrypt data. Once the key has been generated, homomorphic evaluation algorithms operate identically to those in single-key homomorphic encryption. For decryption, at least $t$-out-of-$n$ data contributors need to collaborate to decrypt a ciphertext. In general, it accompanies substantial computational and memory overhead for decryption^12,13^. However, if all $n$ parties come together at the decryption phase (i.e., $t=n$), such overhead is eliminated.

#### The need for flexibility

A significant drawback of the threshold approach is the necessity to predetermine the number of participants before initiating the learning process. In practical scenarios, this is often not feasible due to various challenges.

Firstly, coordinating data collection can be logistically challenging, and not all companies might be ready to begin simultaneously. Some might only be positioned to join after the learning process has already begun. Additionally, there will always be late adopters. Such companies should not be left out and must be given the chance to contribute and benefit from the improved model. On the flip side, some participants may choose to exit the learning process midway. Binding every participant from start to finish feels overly restrictive and impractical. The best approach would be one that seamlessly adapts to such changes without disturbing the ongoing collaboration. In essence, an ideal method should be flexible, accommodate varying timelines for data availability, embrace late participants, and be robust to potential dropouts.

### Our methods

#### Proposed framework

The multi-key framework we introduce in this study sidesteps the constraint of having a set number of participants. It allows future members to join the learning process at any point, thanks to a server-driven mechanism called “ciphertext expansion”^20^. For illustration, suppose Party 1 has $({a}_{1}, {b}_{1})$ as the encryption of ${m}_{1}$, and Party 2 wishes to join with $({a}_{2}, {b}_{2})$ being the encryption of ${m}_{2}$. The central server can expand $({a}_{1}, {b}_{1})$ into $({a}_{1}, {b}_{1}, 0)$ and $({a}_{2}, {b}_{2})$ into $({a}_{2}, 0,{b}_{2})$. This adjustment enables the server to perform homomorphic operations on ciphertexts originating from both parties. For example, the sum of the two expanded ciphertexts, $({a}_{1}+{a}_{2},{b}_{1},{b}_{2})$, results in a valid encryption of ${m}_{1}+{m}_{2}$. This is because ${m}_{1}+{m}_{2}$ can be retrieved by computing $\left({a}_{1}+{a}_{2},{b}_{1},{b}_{2}\right)\cdot \left(1, {s}_{1},{s}_{2}\right)={a}_{1}+{b}_{1}{s}_{1}+{a}_{2}+{b}_{2}{s}_{2}\approx {m}_{1}+{m}_{2}$. For a step-by-step guide on how our multi-key framework achieves homomorphic addition and multiplication across parties, see Supplementary Information S2 and S3.

The benefit of the ciphertext expansion is that the server can smoothly continue the learning as new members join, without burdening the old members with tasks like re-creating keys or sending encrypted data—a hurdle in the earlier threshold method.

**Figure 2:**
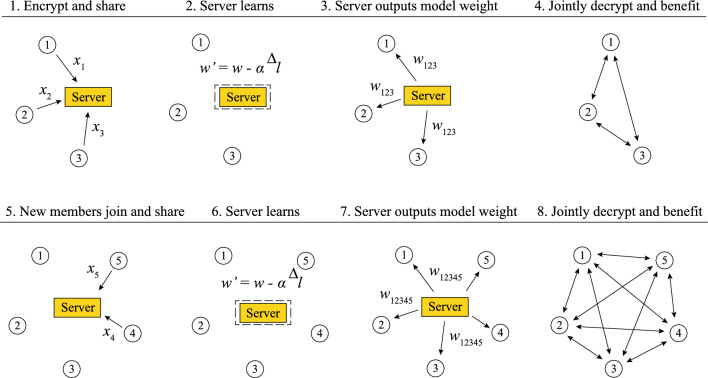
Process of flexible learning with new member integration. The figure outlines an initial learning scenario with three participants (steps 1 to 4), each contributing encrypted data for joint learning. In the following stages (steps 5 to 8), two additional participants seamlessly integrate their data into the existing process. The server initiates ciphertext expansion to incorporate new members into the learning algorithm, leading to the production of refined encrypted weight (${w}_{12345}$) that enhance inference and are returned to all members. Despite the server’s essential role in ciphertext expansion, it remains uninvolved in decryption to ensure the security and privacy of participant data.

More concretely, let’s illustrate a flexible learning scenario using Fig. 2. Initially, for steps 1 to 4, we have three members. Each member creates a secret key, encrypts their data, and sends it to the server. The server expands these ciphertexts, applies the learning algorithm, and outputs $Enc({w}_{123})$. These encrypted weights are relayed back to the members for joint decryption in two stages: partial decryption and merge. During the partial decryption phase, each party uses its secret key to partially decrypt the resulting ciphertext. Subsequently, each party adds some auxiliary noise for security^21^ and shares the partially decrypted ciphertext. In the merge step, members combine all the partially decrypted ciphertexts to derive a plaintext weight vector, enhancing their inference with this refined weight.

Moving on to steps 5 through 8, two additional members join the group. These latecomers generate their own secret keys, encrypt their respective data sets, and send this encrypted information to the server. The server then expands the existing $Enc({w}_{123})$ to accommodate data from the new members. Subsequently, the server continues its online gradient descent calculations, yielding $Enc({w}_{12345})$. This updated weight vector is returned to all members with the expectation that ${w}_{12345}$ will offer improved inference performance over ${w}_{123}$. The benefits are expected to amplify as more participants join the process.

Note that, to accommodate new members, either participants or the server can handle ciphertext expansion; however, for participants’ convenience, the server typically assumes this role. Despite this, the server must not participate in the decryption step for security reasons, as the server could potentially reverse engineer the weight to access sensitive participant data. In essence, the server takes responsibility for expansion for convenience but remains strictly uninvolved in decryption for security.

#### Proposed algorithm: MK-HELR

In this section, we outline the derivation and the implementation of the proposed Multi-Key Homomorphic Encryption Logistic Regression (MK-HELR) algorithm.

##### Logistic regression

First, we lay out the weight update process for logistic regression in our study. We assume independent observations and use Bernoulli to express the likelihood, $L\left(w\right)$, of training data as in Eq. (6), where $y\in \{\text{0,1}\}$ is the binary label and $z={w}^{T}x$ for weight vector $w$ and input $x$.

6 $$L\left( w \right) = { }\mathop \prod \limits_{i = 1}^{n} \sigma \left( {z_{i} } \right)^{{y_{i} }} { }\left\{ {1 - \sigma \left( {z_{i} } \right)} \right\}^{{1 - y_{i} }}$$

Then, the Negative Log Likelihood becomes:

7 $$NLL\left( w \right) = \mathop \sum \limits_{i = 1}^{n} - y_{i} \log \sigma \left( {z_{i} } \right) - \left( {1 - y_{i} } \right)\log \left\{ {1 - \sigma \left( {z_{i} } \right)} \right\}$$

If we apply $\frac{\partial \sigma (z)}{\partial z}=\sigma \left(z\right)\{1-\sigma \left(z\right)\}$, the derivative of $NLL\left(w\right)$ with respect to ${w}_{j}$ becomes:

8 $$\frac{\partial NLL\left( w \right)}{{\partial w_{j} }} = \mathop \sum \limits_{i = 1}^{n} x_{ij} { }\left\{ {\sigma \left( {z_{i} } \right) - y_{i} } \right\}$$

This derivative is used in the gradient descent, which is expressed as:

9 $$w_{j}^{\prime} = w_{j} - \frac{\alpha }{n}\frac{\partial NLL\left( w \right)}{{\partial w_{j} }}$$

Now, we express the term $\frac{\alpha }{n}\frac{\partial NLL\left(w\right)}{\partial {w}_{j}}$ as in Eq. (10), with the aim of keeping the multiplicative depth minimal. For the derivation steps illustrating how the multiplicative depth is reduced, refer to Supplementary Information S4. Here, $\sigma \left(x\right)\approx {c}_{0}+{c}_{1}x+{c}_{3}{x}^{3}$ is used, where $\left( {c_{0} ,c_{1} ,c_{3} } \right) = \left( {0.5,{ }\frac{1.20096}{8},{ } - \frac{0.81562}{{8^{3} }}} \right)$.

10 $$\frac{\alpha }{n}\frac{\partial NLL\left( w \right)}{{\partial w_{j} }} = \mathop \sum \limits_{i = 1}^{n} \left( {{ }\frac{\alpha }{n}c_{3} x_{ij} z_{i} } \right){*}\left( {z_{i}^{2} + \frac{{c_{1} }}{{c_{3} }}} \right) + \frac{\alpha }{n}\left( {c_{0} - y_{i} } \right)x_{ij}$$

For weight updates, we apply the Nesterov’s Accelerated Gradient (NAG) algorithm^22^, as outlined in Eq. (11) and (12), achieving a convergence rate of $O(\frac{1}{{k}^{2}})$, unlike the standard $O\left(\frac{1}{k}\right)$ rate. A brief overview of this method is available in Supplementary Information S.5.

11 $$\beta_{k + 1} = w_{k} - \frac{\alpha }{n}\frac{{\partial NLL(w_{k} )}}{{\partial w_{k} }}$$

12 $$w_{k + 1} = \left( {1 - \gamma_{k} } \right)\beta_{k + 1} + \gamma_{k} \beta_{k}$$

Here, ${\beta }_{0}$ is equal to ${w}_{0}$, and ${\gamma }_{k}$ is defined in the equation below.

13 $$\gamma_{k} = \frac{{1 - \lambda_{k} }}{{\lambda_{k + 1} }},{ }\lambda_{k} = \frac{{1 + \sqrt {1 + 4\lambda_{k - 1}^{2} } }}{2} \left( {\lambda_{0} = 0} \right)$$

##### MK-HELR algorithm

**Figure 3:**
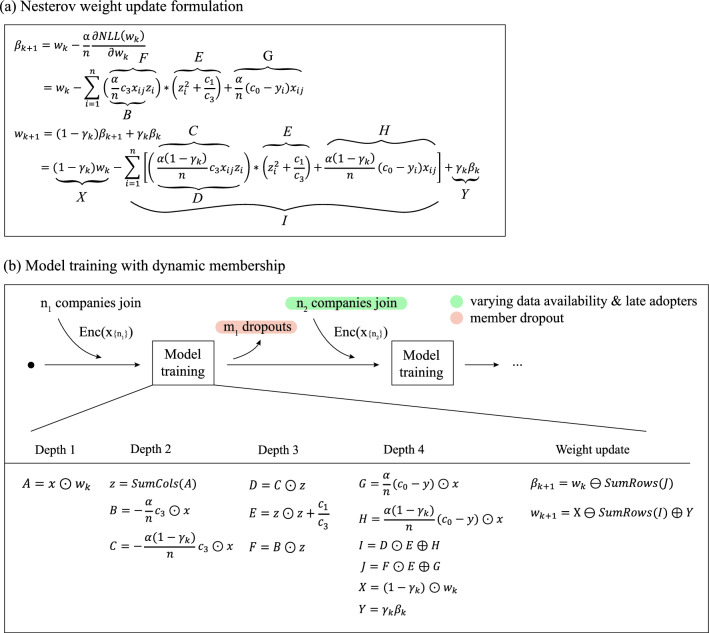
Schematic of the MK-HELR Algorithm. (**a**) Nesterov’s accelerated gradient descent formulation is presented, where the consumption of multiplicative depth is limited to only four per training iteration. (**b**) The flexibility of the MK-HELR algorithm is highlighted, showcasing its ability to incorporate new members and allow member departures at any given point without disturbing existing parties. This visualization emphasizes the algorithm’s capacity to manage fluctuating data timelines and evolving memberships, all the while ensuring data privacy.

Next, we outline the MK-HELR (Multi-Key Homomorphic Encryption Logistic Regression) algorithm, capable of performing logistic regression training on encrypted aggregate data contributed by multiple entities. An essential step is depicted in Fig. 3a, where we restructure the Nesterov’s accelerated gradient descent formulation so that only four multiplicative depths^23^ are consumed per iteration during training. This optimization is crucial; the fewer depths consumed per iteration, the more iterations we can perform. This is because the multiplicative depth is constrained by the number of primes used for the ciphertext modulus in our setting. The derivation details of this depth consumption minimization are available in Supplementary Information S4.

Next, Fig. 3b demonstrates how the MK-HELR algorithm can seamlessly incorporate new member data at any stage of the learning process, without demanding any action from existing parties, all while ensuring data privacy for every participant. Specifically, it shows model training based on the initial ${n}_{1}$ companies, then a second round of learning (weight update) after ${m}_{1}$ companies depart and ${n}_{2}$ companies join. To reiterate, the ability to integrate this new data is achieved through ciphertext expansion. We emphasize that this diagram underscores our algorithm's capacity to adapt to fluctuating data availability timelines, encourage inclusion for those who join later, and remain robust against member dropout.

#### Algorithm implementation

The essential tools for the proposed dynamic and flexible learning environment have been built using the Lattigo library^24^ under the multi-key CKKS scheme^16,20^, as summarized:

- MK-CKKS.KeyGen($${\varvec{i}}$$): Each party $$i$$ generates a tuple consisting of a secret key, encryption key, and evaluation key ($$s{k}_{i},p{k}_{i}, ev{k}_{i})$$. The encryption key and evaluation key are publicly broadcasted to other parties.
- MK-CKKS.Enc(${\varvec{p}}{{\varvec{k}}}_{{\varvec{i}}};{\varvec{\mu}}$): Given an encryption key $p{k}_{i}$ and a message $\mu$, the output is a ciphertext $ct$ belonging to ${R}_{{Q}_{L}}^{2}$ and the index set of the corresponding parties $I: = \left\{ i \right\}$.
- MK-CKKS.Expand($\left( {{\varvec{ct}},{\varvec{I}}} \right),{\varvec{I}}^{\prime } )$: Given a ciphertext $ct=\left({c}_{0},{\left\{{c}_{i}\right\}}_{i\in I}\right)\in {R}_{{Q}_{{\ell}}}^{\left|I\right|+1}$ and the index set $I^{\prime } \supset I$, output the ciphertext $\overline{ct} = \left( {c_{0} ,\left\{ {c_{i} } \right\}_{{i \in I{^{\prime}}}} } \right) \in R_{{Q_{\ell } }}^{{\left| {I^{\prime } } \right| + 1}}$ where $c_{i} : = 0$ for $i \in I^{\prime } \backslash I$. If $I = I^{\prime }$, then $\overline{ct }=ct.$
- MK-CKKS.Add($\left( {{\varvec{ct}},{\varvec{I}}} \right),\left( { {\varvec{ct}}^{\prime } , {\varvec{I}}^{\prime } } \right))$: Set $I_{add} : = I \cup I^{\prime}$, and expand the input ciphertexts $ct\in {R}_{{Q}_{{\ell}}}^{\left|I\right|+1}$ and $ct^{\prime } \in R_{{Q_{l} }}^{{\left| {I^{\prime } } \right|}}$ as $\overline{ct }\leftarrow$ MK-CKKS.Expand($(ct,I)$, ${I}_{add}$) and as $\overline{{ct^{\prime } }} \leftarrow$ MK-CKKS.Expand($\left( {ct^{\prime } ,I^{\prime } } \right)$, ${I}_{add}$). Output a pair of the ciphertext $ct_{add} : = \overline{ct} + \overline{{ct^{\prime } }} \in R_{{Q_{\ell } }}^{{\left| {I_{add} } \right| + 1}}$ and the corresponding index set ${I}_{add}$.
- MK-CKKS.Mult$$(\left\{ {{\varvec{pk}}_{{\varvec{i}}} } \right\}_{{{\varvec{i}} \in {\varvec{I}} \cup {\varvec{I}}^{\prime } }} ;\left( {{\varvec{ct}},{\varvec{I}}} \right),\left( { {\varvec{ct}}^{\prime } , {\varvec{I}}^{\prime } } \right))$$: Set $$I_{mult} : = I \cup I^{\prime }$$, and expand the input ciphertexts $$ct\in {R}_{{Q}_{{\ell}}}^{\left|I\right|+1}$$ and $$ct^{\prime } \in R_{{Q_{\ell } }}^{{\left| {I^{\prime } } \right| + 1}}$$ as $$\overline{ct }\leftarrow$$ MK-CKKS.Expand($$(ct,I)$$, $${I}_{mult}$$) and as $$\overline{{ct^{\prime } }}$$$$\leftarrow$$ MK-CKKS.Expand($$\left( {ct^{\prime } ,I^{\prime } } \right)$$, $$I_{mult}$$). From $$\overline{ct }$$ and $$\overline{{ct^{\prime } }}$$, compute and output the ciphertext $$c{t}_{mult}\in {R}_{{Q}_{{\ell}}}^{|{I}_{mult}|+1}$$ and the corresponding index set $${I}_{mult}$$.
- MK-CKKS.Rescale($({\varvec{c}}{\varvec{t}},{\varvec{I}}))$: Given a ciphertext $ct=\left({c}_{0},{\left\{{c}_{i}\right\}}_{i\in I}\right)\in {R}_{{Q}_{{\ell}}}^{\left|I\right|+1}$, output $ct^{\prime } = { }\left( {c_{0}^{{^{\prime } }} ,\left\{ {c_{i}^{{^{\prime } }} } \right\}_{i \in I} } \right) \in R_{{Q_{\ell - 1} }}^{\left| I \right| + 1}$ where $c_{i}^{{^{\prime } }} : = q_{\ell }^{ - 1} \cdot c_{i} \in R_{{Q_{\ell - 1} }}$ for $i\in \left\{0\right\}\cup I$.
- MK-CKKS.Dec(${\left\{{\varvec{s}}{{\varvec{k}}}_{{\varvec{i}}}\right\}}_{{\varvec{i}}\in {\varvec{I}}};({\varvec{c}}{\varvec{t}},{\varvec{I}}))$: Given a ciphertext  $ct=\left({c}_{0},{\left\{{c}_{i}\right\}}_{i\in I}\right)\in {R}_{{Q}_{{\ell}}}^{|I|+1}$ and the associated secret keys ${\left\{s{k}_{i}\right\}}_{i\in I}$, return $\mu : = c_{0} + \mathop \sum \limits_{i \in I} c_{i} \cdot sk_{i}$ (mod ${Q}_{{\ell}})$.

## Results and discussion

### MK-HELR performance evaluation

In this section, we test our proposed method using the AI4I, a publicly available predictive maintenance dataset^18^. We divide it into five subsets via random sampling, assigning data to four companies with one subset reserved as a held-out test dataset. Each feature in the subsets is standardized, using the formula $\frac{x-\mu }{\sigma }$. The subsets are then encrypted. For encryption, we used a ring dimension of ${2}^{15}$ and a ciphertext modulus of 810 bits. CKKS configuration settings include a scaling factor of ${2}^{38}$, a Gaussian distribution for error, and a ternary distribution for the secret key. We employed the RNS gadget decomposition using base values of 38-bit size primes.

**Figure 4:**
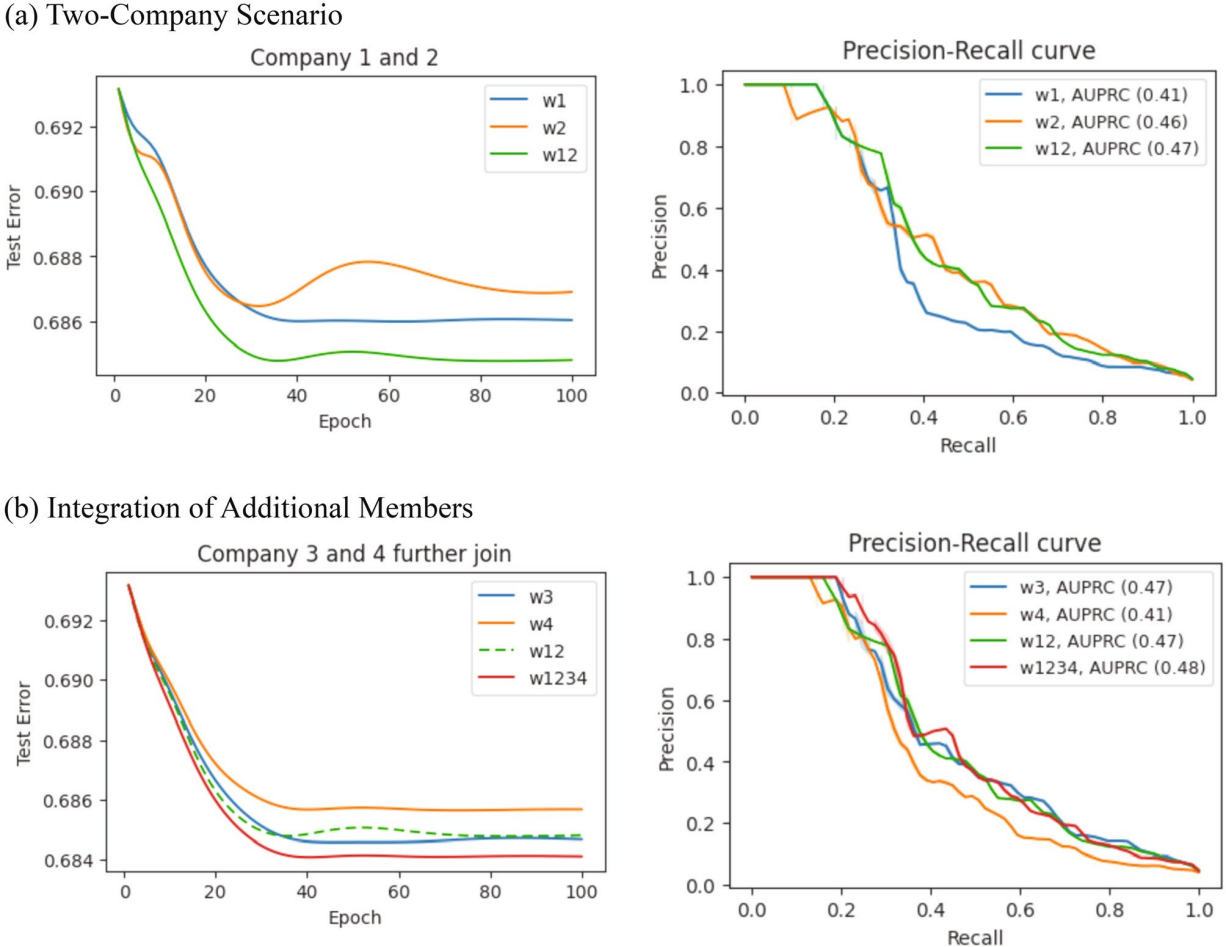
Enhanced Performance with MK-HELR Algorithm’s Collaborative Learning. (**a**) The MK-HELR algorithm merges encrypted data from two companies, resulting in reduced test error and an enhanced AUPRC compared to individual learning. (**b**) Extending from the initial two-company scenario, the algorithm incorporates additional data from another two companies. The reduced test error and enhanced AUPRC in this four-company interaction emphasizes the merit of flexible, collaborative learning.

Figure 4 presents the learning process of our MK-HELR algorithm on the encrypted data provided by the different parties. For these experiments, the learning rate ($\alpha$) is set to 0.3, the weight vector ($w$) is initialized with zeros, and the negative log likelihood is used as the loss function. Each subscript of $w$ in the figure corresponds to the companies whose data are used in deriving that weight. For example, ${w}_{12}$ is derived from the data of companies 1 and 2.

Figure 4a depicts a scenario where the MK-HELR algorithm trains a logistic regression model using encrypted data from two companies. The collaborative learning approach yields lower test errors and a higher AUPRC (Area Under the Precision-Recall Curve), a key metric for classification models, compared to learning from individual datasets. In Fig. 4b, the MK-HELR algorithm allows for the addition of new members during the learning process, a noteworthy improvement over traditional methods that require a set number of participants at the start. As more data from new members are integrated, the model’s performance improves, indicated by the decrease in test errors and the increase in AUPRC.

A critical aspect of our analysis of the results presented in Figs. 4a and b pertains to the number of training cycles, or epochs. These epochs necessitate a corresponding number of multiplicative depths due to the multiplicative computations involved in each epoch. Multiplicative depths indicate how often an encrypted data piece can be multiplied before needing decryption. In our case, when the depths were exhausted, we manually decrypted and re-encrypted the data to replenish them.

Upon close examination, it becomes apparent that a more efficient strategy might be to expand the *bootstrapping* method^25^. This cryptographic procedure minimizes ciphertext noise produced by homomorphic operations and resets depths by homomorphically evaluating the decryption circuit. Many studies have tackled bootstrapping in single-key HE contexts^26–28^. Yet, it is challenging to directly apply this to multi-key scenarios due to the accelerated noise increase in multi-key settings. Therefore, we suggest that future research might focus on redefining cryptographic parameters via error analysis.

Moving on to computation time, our experiments provide an insightful perspective on our algorithm's proportional scaling with the number of parties involved. Specifically, the computation time was 317 s for four parties, roughly twice the 159 s for two parties, and 82 s for a single party, nearly a fourth of the time for four parties. These tests were performed on a machine equipped with an Intel Xeon Platinum 8268 2.90 GHz CPU and 192 GB RAM running Ubuntu 20.04.2 LTS. This linear increase in computation time directly relates to each party independently creating their own secret or public key, a key feature of our algorithm. This unique requirement separates our approach from other methods, which are further analyzed in detail in “Comparison with other algorithms”.

At the cost of linearly increasing computational complexity, our algorithm provides exceptional flexibility and dynamism. It allows any group of parties to collaborate on model training and enables new parties to join the computation without disrupting the ongoing process. This feature aligns closely with real-world scenarios where adding new participants should ideally not interrupt the learning procedure or the existing parties. Note that the MK-HELR algorithm does not guarantee an improved weight vector; instead, it hints at the possibility of such an improvement, firmly grounded in established statistical learning theories.

### Comparison with other algorithms

We compare our MK-HE algorithm with other key algorithms, focusing on dynamic membership, computation cost, and the need for a trusted third party—three core aspects for collaborative learning settings. The results of this comparative analysis are summarized in Table 1, with further details for each algorithm provided in the subsequent text.

**Table 1:** Comparative analysis of single-key, threshold, and multi-key HE algorithms.

	Single-key HE	Threshold HE	Multi-key HE
Dynamic membership	O	X	O
Computation cost (with respect to number of parties, $k$)	$O(1)$	$O(1)$	$O(k)$
Need for trusted third party	O	X	X

#### Single-key HE

In developing a privacy-preserving collaborative learning algorithm for multiple parties, Single-Key Homomorphic Encryption (Single-Key HE)^23^ can be an initial consideration. This technique presumes that each participant in the computation uses the same public key for encryption or evaluation.

This shared key arrangement allows for any party to either join or exit the computation process at different points—enabling dynamic membership—since all share the same public key. It also ensures the computation cost for collaborative learning does not depend on the number of parties involved as the computations proceed using one shared public key.

However, this setup requires a trusted third party in possession of the secret key to distribute the public key to all collaborators who lack access to the secret key. Without the trusted third party, any party holding the secret key could decrypt others’ ciphertexts, posing a security risk. The requirement of such a trustworthy entity is often unrealistic in practical scenarios, making Single-Key HE less viable in multi-party contexts. If such a trusted party existed, cryptographic tools for privacy-preserving computations would be unnecessary due to the inherent trust. Challenging this unrealistic assumption, studies have proposed solutions like Threshold HE and Multi-Key HE.

#### Threshold HE

Threshold Homomorphic Encryption (Th-HE) requires multiple parties to work together to generate a joint public key used for encryption^13,19,29^, setting it apart from the Single-Key HE where a trusted third party dispatches a public key to all participants. The computational complexity of Th-HE is a lightweight $O(1)$, relative to the number of parties, mirroring the complexity of Single-Key HE. This similarity arises because the shared key in Th-HE operates similarly to the Single-Key HE’s public key—only this joint key is used in the computation.

However, Th-HE carries a substantial functional limitation. It necessitates a predetermined participant list during the key generation phase and requires all parties’ presence to construct the shared key. Consequently, Th-HE does not permit the on-the-fly addition of new members during the collaborative homomorphic computation.

The only feasible, yet practically challenging approach, involves all parties having to reinitiate the configuration process every time new members join. This necessity poses considerable operational challenges and requires additional rounds of communication from a computational perspective. Notably, in real-world applications, this limitation means that any time a new member is introduced, complexities in terms of coordination and communication with all existing collaborating entities arise, which can cause significant delays, disrupting the continuity of the learning process and the collaborative benefit for the remaining participants.

#### Multi-key HE

Our algorithm is primarily centered on the Multi-Key Homomorphic Encryption (MK-HE) method, offering superior flexibility and dynamism. This approach allows each collaborating party to generate its respective encryption key independently. It supports collaboration on model training among any group of parties and facilitates the inclusion of new parties to the computation at any point. These features ensure the ongoing learning process remains undisturbed throughout. This consideration closely aligns with real-world scenarios where ideally, the addition of a new participant should not disrupt the existing parties or the ongoing learning procedure.

One limitation of MK-HE is its computational complexity, which increases linearly with the number of engaged parties, as outlined in Table 1. This complexity arises because each party generates its secret/public keys independently, as opposed to the uniform key generation in Single-Key HE or Th-HE setups. Consequently, the computational complexity in MK-HE is directly proportional to the number of involved parties, whereas it remains constant in both Single-Key HE and Th-HE setups^20^.

Despite this drawback, the unique capability of MK-HE to dynamically onboard new parties during any intermediary stage of the collaborative homomorphic computation effectively closes this *functional gap* and makes it highly suitable for various practical scenarios. Specifically, MK-HE is an optimal choice in situations requiring models that can continuously accommodate new data sources without delays, adapt to participant transitions effortlessly, and ensure a disruption-free learning experience for all parties involved.

## Extensibility and limitations

### Extensibility

#### Collaborative and competitive frameworks

The MK-HELR algorithm can be applied in various collaborative and competitive frameworks, where entities pursue shared objectives but also compete in various dimensions. For example, airlines jointly aim to minimize flight delays, yet they use competitive strategies based on private disruption management information^30^. Likewise, Uber and Lyft both want efficient city transportation, like effective traffic management systems, but they compete for rides based on proprietary pricing data^31^. Retailers strive to enhance their supply chains but face competition due to confidential information regarding order quantities and contract terms^32^. These challenges are traditionally tackled through sophisticated contracts or mechanisms designed to align behaviors among agents^33^. Our algorithmic approach may offer a potential solution to such long-standing problems.

#### Multi-key homomorphic encryption support vector machines

From a theoretical perspective, we note that the loss function used in the Support Vector Machine (SVM) is also convex and Lipschitz^34^. Consequently, future studies could explore the implementation of a multi-key homomorphic encryption support vector machine (MK-HESVM) algorithm, following the same principles as our approach. The strengths of the SVM, such as its capability to handle high-dimensional data^35^, make the development of the MK-HESVM a promising endeavor.

### Limitations

#### Reduction in the upper bound of rademacher complexity

While our method offers a reduction in the upper bound of Rademacher Complexity, it is essential to recognize that this reduction has its limits. As data size grows beyond a certain threshold, we might not observe further benefits. Intuitively, there is often a ceiling to the amount of information the available data can capture.

#### Scalability

The presented MK-HELR algorithm demonstrates the advantages of secure and adaptable collaborative learning. However, it is crucial to evaluate the scalability of the algorithm as the number of participants increases. It is worth noting that the algorithm is constrained by a limited multiplicative depth, primarily because of the restrictions on the number of primes used for the ciphertext modulus to ensure security. Future research may focus on enhancing the arithmetic circuits of multi-party algorithms to better accommodate larger-scale scenarios and subsequently test its performance under those conditions.

Considering these extensibility and limitations, we anticipate future research to build upon our work and further refine the proposed framework for broader applicability across diverse domains and settings involving collaborative and competitive frameworks.

## Conclusion

In conclusion, this study introduces a novel collaborative learning framework with dynamic membership, leveraging multi-key homomorphic encryption for secure and adaptable joint learning. Our work highlights the benefits of learning from aggregate data from the standpoint of statistical learning and argues for the necessity of a flexible and dynamic framework that can seamlessly integrate new members and their data. The proposed MK-HELR (multi-key homomorphic encryption logistic regression) algorithm addresses data privacy concerns while accommodating a range of data availability scenarios, reflecting real-world conditions. Our demonstration of the algorithm on a publicly available predictive maintenance dataset showcases its potential and broad applicability. Furthermore, our discussion on the extensibility of the method to industrial frameworks involving both collaboration and competition underscores the wider impact and relevance of our work. We believe that this study lays the groundwork for future research focused on implementing various learning algorithms with dynamic membership, thereby fostering more secure, inclusive, and resilient collaborative learning environments.

## Supplementary Information

Supplementary Information.

## Data Availability

The datasets examined in this study are accessible in the UCI Machine Learning Repository^18^, located at 10.24432/C5HS5C.

## List of symbols

$$x$$: Input features
$$y$$: Target label
$$f$$: Classification model
$n$: Number of observations
MK-HELR: Multi-Key Homomorphic Encryption Logistic Regression
Margin: $yf(x)$in a classification problem
$l$: Margin loss function
$$L$$: Expected risk
$$\widehat{L}$$: Empirical risk
$w$: Model parameter (weight vector)
$$\lambda$$: Regularization parameter
$\widehat{{w}_{\lambda }}$: Solution of regularized empirical risk minimization
$\mathcal{V}\left(\lambda \right)$: Estimation error (sample error)
$$Rad$$: Rademacher complexity
$${\varepsilon }_{i}$$: Rademacher variable$$\pm 1$$
$${C}_{L}$$: Lipschitz constant
$$\widehat{y}$$: Model prediction
$$\sigma$$: Sigmoid function
$$\alpha$$: Learning rate
$\nabla l$: Gradient of loss function
$NLL$: Negative Log Likelihood
$\odot$: Entry-wise multiplication
$\oplus$: Entry-wise addition
$\ominus$: Entry-wise subtraction

## Code availability

The custom code used in this research is publicly available on GitHub at https://github.com/hdavidkang/share2gain. The version of the code that supports the findings of this study has been archived and can be accessed via 10.5281/zenodo.10928477.

## References

[1] Data sharing in the age of deep learning. *Nat. Biotechnol.* **41**, 433-433 (2023). 10.1038/s41587-023-01770-3 37020134

[2] Mohri, M., Rostamizadeh, A. & Talwalkar, A. *Foundations of Machine Learning* (MIT Press, 2018).

[3] Quach, S., Thaichon, P., Martin, K. D., Weaven, S. & Palmatier, R. W. Digital technologies: Tensions in privacy and data. *J. Acad. Mark. Sci.* **50**, 1299–1323 (2022). 35281634 10.1007/s11747-022-00845-y PMC8897618

[4] Farayola, O. A., Olorunfemi, O. L. & Shoetan, P. O. Data privacy and security in IT: A review of techniques and challenges. *Comput. Sci. IT Res. J.* **5**, 606–615 (2024).

[5] Beltrán, E. T. M. *et al.* Decentralized federated learning: Fundamentals, state of the art, frameworks, trends, and challenges. *IEEE Commun. Surv. Tutor.* **25**(4), 2983–3013 (2023).

[6] Sheller, M. J. *et al.* Federated learning in medicine: Facilitating multi-institutional collaborations without sharing patient data. *Sci. Rep.* **10**, 12598 (2020). 32724046 10.1038/s41598-020-69250-1 PMC7387485

[7] Khan, L. U. *et al.* Resource optimized federated learning-enabled cognitive internet of things for smart industries. *IEEE Access* **8**, 168854–168864 (2020).

[8] Bagheri, B., Rezapoor, M. & Lee, J. A unified data security framework for federated prognostics and health management in smart manufacturing. *Manuf. Lett.* **24**, 136–139 (2020).

[9] Shokri, R., Stronati, M., Song, C. & Shmatikov, V. Membership inference attacks against machine learning models. *2017 IEEE symposium on security and privacy*, 3–18 (2017).

[10] Geiping, J., Bauermeister, H., Dröge, H. & Moeller, M. Inverting gradients-how easy is it to break privacy in federated learning?. *Adv. Neural Inf. Process. Syst.* **33**, 16937–16947 (2020).

[11] Gentry, C. Computing arbitrary functions of encrypted data. *Commun. ACM* **53**, 97–105 (2010).

[12] Mouchet, C., Bertrand, E. & Hubaux, J.-P. An efficient threshold access-structure for rlwe-based multiparty homomorphic encryption. *J. Cryptol.* **36**, 10 (2023).

[13] Boneh, D. et al. Threshold cryptosystems from threshold fully homomorphic encryption. *Advances in Cryptology–CRYPTO 2018: 38th Annual International Cryptology Conference, Santa Barbara, CA, USA, August 19–23, 2018, Proceedings, Part I 38*, 565–596 (2018).

[14] Boudgoust, K. & Scholl, P. *Simple Threshold (Fully Homomorphic) Encryption from LWE with Polynomial Modulus* (Springer, 2023).

[15] Kang, H. E. D. *et al.* Homomorphic encryption as a secure PHM outsourcing solution for small and medium manufacturing enterprise. *J. Manuf. Syst.* **61**, 856–865 (2021).

[16] Chen, H., Dai, W., Kim, M. & Song, Y. Efficient multi-key homomorphic encryption with packed ciphertexts with application to oblivious neural network inference. *Proceedings of the 2019 ACM SIGSAC Conference on Computer and Communications Security*, 395–412 (2019).

[17] Chen, H., Chillotti, I. & Song, Y. *Multi-key homomorphic encryption from TFHE* (Springer, 2019).

[18] UCI Machine Learning Repository, AI4I 2020 Predictive Maintenance Dataset. 10.24432/C5HS5C (2020).

[19] Mouchet, C., Troncoso-Pastoriza, J., Bossuat, J. P. & Hubaux, J. P. Multiparty homomorphic encryption from ring-learning-with-errors. *Proc. Priv. Enhanc. Technol.* **2021**, 291–311 (2021).

[20] Kim, T., Kwak, H., Lee, D., Seo, J. & Song, Y. Asymptotically faster multi-key homomorphic encryption from homomorphic gadget decomposition. *Proceedings of the 2023 ACM SIGSAC Conference on Computer and Communications Security*, 726–740 (2023).

[21] Dahl, M. *et al.* Noah’s Ark: Efficient Threshold-FHE Using Noise Flooding. *Proceedings of the 11th Workshop on Encrypted Computing & Applied Homomorphic Cryptography*, 35–46 (2023).

[22] Nesterov, Y. A method of solving a convex programming problem with convergence rate O (1/k** 2). *Dokl. Akad. Nauk SSSR* **269**, 543 (1983).

[23] Crockett, E. A low-depth homomorphic circuit for logistic regression model training. *Cryptology ePrint Archive* (2020).

[24] EPFL-LDS. *Lattigo v2.3.0.* https://github.com/ldsec/lattigo (2021).

[25] Gentry, C. Fully homomorphic encryption using ideal lattices. *Proceedings of the forty-first annual ACM symposium on Theory of computing*, 169–178 (2009).

[26] Cheon, J. H., Han, K., Kim, A., Kim, M. & Song, Y. Bootstrapping for approximate homomorphic encryption. *Advances in Cryptology–EUROCRYPT 2018: 37th Annual International Conference on the Theory and Applications of Cryptographic Techniques, Tel Aviv, Israel, April 29-May 3, 2018 Proceedings, Part I 37*, 360–384 (2018).

[27] Han, K. & Ki, D. *Better Bootstrapping for Approximate Homomorphic Encryption* (Springer, 2020).

[28] Lee, J.W., Lee, E., Lee, Y., Kim, Y.S. & No, J.S. High-precision bootstrapping of RNS-CKKS homomorphic encryption using optimal minimax polynomial approximation and inverse sine function. *Advances in Cryptology–EUROCRYPT 2021: 40th Annual International Conference on the Theory and Applications of Cryptographic Techniques, Zagreb, Croatia, October 17–21, 2021, Proceedings, Part I 40*, 618–647 (2021).

[29] Asharov, G. *et al.* Multiparty computation with low communication, computation and interaction via threshold FHE. *Advances in Cryptology–EUROCRYPT 2012: 31st Annual International Conference on the Theory and Applications of Cryptographic Techniques, Cambridge, UK, April 15–19, 2012. Proceedings 31*, 483–501 (2012).

[30] Wu, C. L. *Airline Operations and Delay Management: Insights from Airline Economics, Networks and Strategic Schedule Planning* (Routledge, 2016).

[31] MacKay, A. & Weinstein, S. N. Dynamic pricing algorithms, consumer harm, and regulatory response. *Wash. UL Rev.* **100**, 111 (2022).

[32] Bart, N., Chernonog, T. & Avinadav, T. Revenue-sharing contracts in supply chains: A comprehensive literature review. *Int. J. Prod. Res.* **59**, 6633–6658 (2021).

[33] Huang, Y., Han, W. & Macbeth, D. K. The complexity of collaboration in supply chain networks. *Supply Chain Manag. Int. J.* **25**, 393–410 (2020).

[34] Geoffrey, C., Guillaume, L. & Matthieu, L. Robust high dimensional learning for Lipschitz and convex losses. *J. Mach. Learn. Res.* **21**, 1–47 (2020).34305477

[35] Ray, P., Reddy, S. S. & Banerjee, T. Various dimension reduction techniques for high dimensional data analysis: A review. *Artif. Intell. Rev.* **54**, 3473–3515 (2021).

