# Supplementary material for: Harnessing the potential of shared data in a secure, inclusive, and resilient manner via multi-key homomorphic encryption
Source: Sci Rep. 2024 Jun 13;14:13626. doi: 10.1038/s41598-024-63393-1 (PMC11637100; doi:10.1038/s41598-024-63393-1)
Supplement: Supplementary file 1 — Supplementary Information. [file 41598_2024_63393_MOESM1_ESM.docx]

Supplementary Information

**S.1. Principle of Homomorphic Encryption**

*Principle of Homomorphic Encryption*

For readers who are encountering Homomorphic Encryption (HE) for the first time, we will attempt to explain how HE operates in the simplest way possible. Regrettably, the abundance of introductory materials online often begins with a heavy use of abstract algebraic notations, causing those unfamiliar with the domain to lose interest. Consequently, we will explain using intuitive examples that focus only on the key concepts, making it easier to follow.

The core question we should ask is:

*How can we encrypt a vector of real numbers such that an external entity cannot decipher it back to its original, yet arithmetic operations remain feasible on the encrypted data?*

The process of mapping (encrypting) a vector of real numbers to a ciphertext is comprised of two steps. The first step is *encoding*. This phase involves expressing a vector of real numbers as a polynomial.

For instance, take the polynomial $d_{0}+d_{1}x^{1}+d_{2}x^{2}$. We determine the coefficients, $d_{i}$, such that when a specific number, say $\xi$, is substituted into this polynomial, it yields the original number $z$. That is, if I have three real numbers, specifically $z_{1}, z_{2}$, and $z_{3}$, then the coefficients $(d_{0}, d_{1}, d_{2})$ for $m\left( x \right)=d_{0}+d_{1}x^{1}+d_{2}x^{2}$ satisfy the condition $m\left( x \right)\left. \right|_{x=\xi_{i}}=z_{i}$. In other words, equation S.1.1 holds.

| $\left[ \begin{matrix} 1 & \xi_{0} & \xi_{0}^{2} \\ 1 & \xi_{1} & \xi_{1}^{2} \\ 1 & \xi_{2} & \xi_{2}^{2} \end{matrix} \right]\left[ \begin{matrix} d_{0} \\ d_{1} \\ d_{2} \end{matrix} \right]=\left[ \begin{matrix} z_{0} \\ z_{1} \\ z_{2} \end{matrix} \right]$ | S.1.1 |
| --- | --- |

Notably, the matrix on the left is structured using geometric progressions and is referred to as the *Vandermonde* matrix in mathematics. If $\xi_{i}$ is chosen as a root of a cyclotomic polynomial, i.e., $\Phi_{n}\left( x \right)=\prod_{\gcd\left( k,n \right)=1} (x-e^{2i\pi\frac{k}{n}})$ for $1\leq k\leq n$, where the roots of this polynomial are the $n$^th^ primitive roots of unity, we can see that a compute acceleration can be achieved in the style of the Fast Fourier Transform.

When multiplied by the matrix inverse on each side of equation S.1.1, the encoding process can be represented as in equation S.1.2; it can be considered that $(z_{0}, z_{1}, z_{2})$ is encoded into the coefficients of the polynomial $(d_{0}, d_{1}, d_{2})$.

| $\left[ \begin{matrix} 1 & \xi_{0} & \xi_{0}^{2} \\ 1 & \xi_{1} & \xi_{1}^{2} \\ 1 & \xi_{2} & \xi_{2}^{2} \end{matrix} \right]^{-1}\left[ \begin{matrix} z_{0} \\ z_{1} \\ z_{2} \end{matrix} \right] \overset{\to}{encoding} \left[ \begin{matrix} d_{0} \\ d_{1} \\ d_{2} \end{matrix} \right]$ | S.1.2 |
| --- | --- |

Following the encoding step, the *encryption* step comes next. Here, the polynomial $m\left( x \right)$ is mapped, as shown in equation S.1.3, to a pair of polynomials $\left( c_{0}, c_{1} \right)$, which represents the ciphertext of the original message $m$.

| $\left( c_{0}, c_{1} \right)=\left( m,0 \right)+r\left( b,a \right)+\left( e_{0}, e_{1} \right)$ | S.1.3 |
| --- | --- |

- $\left( m,0 \right)$ is the polynomial message from the previous encoding step.
- $r$ is a temporary secret key used to encrypt $m$ with a public key $(b,a)$.
- $(b,a)$ is the public key that satisfies $b=-as+e$, where s is the secret key and $e$ is a small noise drawn from a Gaussian distribution.
- $e_{0}$ and $e_{1}$ are small noise, independently drawn from a Gaussian distribution.

Simply put, this process is essentially encrypting $m$ using the public key, while introducing a bit of noise. Though it may seem bizarre at first glance, its elegance is evident in three key aspects.

Firstly, the tuple $\left( c_{0}, c_{1} \right)$ can be decrypted back to the original message $m$, simply by executing a dot product with the tuple $\left( 1, s \right)$ as demonstrated in equation S.1.4. This results in $m$ plus some small term, returning the original message $m$ with a minor noise component. Importantly, this decryption is conditional on the knowledge of $s$, thereby restricting this capability to the owner of the secret key.

| $\left( c_{0}, c_{1} \right)\cdot\left( 1, s \right)=\left( m,0 \right)\cdot\left( 1, s \right)+r\left( b,a \right)\cdot\left( 1, s \right)+\left( e_{0}, e_{1} \right)\cdot\left( 1, s \right)$  $=m+r\left( b+as \right)+e_{0}+e_{1}s=m+re+e_{0}+e_{1}s=m+small$ | S.1.4 |
| --- | --- |

Secondly, this representation retains the ability to perform arithmetic operations like addition. For instance, the sum of two ciphertexts is equivalent to the encryption of the sum of their corresponding messages, as demonstrated in equation S.1.5. This confirms the homomorphic property, maintaining the arithmetic operability across both the plain (unencrypted) and encrypted domains. However, unlike additions, operations such as “$>$” in the encrypted domain seem non-trivial. We will address such comparison operators later in the thesis.

| $\left( c_{0}+c_{0}^{'}, c_{1}+c_{1}' \right)\cdot\left( 1, s \right)=m_{1}+m_{2}+small$ | S.1.5 |
| --- | --- |

Thirdly, the introduction of the noise term $\left( e_{0}, e_{1} \right)$ ensures that the ciphertext $\left( c_{0}, c_{1} \right)$ can only be deciphered by someone possessing the secret key, effectively concealing the original message $m$ from unauthorized parties. This level of security is guaranteed by the Learning With Errors (LWE) framework ^1^, a contemporary standard of cryptographic hardness assumption. Introduced by Regev in 2005, LWE has been proven to be as difficult to solve as some of the most intractable lattice problems in the field of mathematics ^2^. Thanks to the LWE hardness assumption, protection of both the secret key and the message is guaranteed.

**S.2. Multi-Key Homomorphic Addition**

To explain how addition is achieved in the Multi-Key CKKS scheme, we provide an illustrative example.

**Step 1.** Two-Party Data Addition

Party 1 creates a ciphertext $ct:=\left( c_{0},c_{1} \right)\in R_{Q}^{2}$ and the index set $I_{1}=\{1\}$. Similarly, Party 2 produces a ciphertext $ct':=\left( c_{0}^{'},c_{1}^{'} \right)\in R_{Q}^{2}$ with the index set $I_{2}=\{2\}$. To homomorphically add $ct$ and $ct'$, the server expands them to $\bar{ct}=(c_{0},c_{1},0)$ and $\bar{ct'}=(c_{0}^{'},0,c_{1}^{'})$. It then outputs the ciphertext ${ct}_{add12}=\bar{ct}+\bar{ct'}=\left( c_{0}+c_{0}^{'},c_{1},c_{1}^{'} \right)\in R_{Q}^{3}$and the combined index set $I_{1,2}:=I_{1}\cup I_{2}=\{1,2\}$.

**Step 2.** Incorporating Party 3

Suppose Party 3 wants to join. This party generates a ciphertext $ct'':=\left( c_{0}^{''},c_{1}^{''} \right)\in R_{Q}^{2}$ and the index set $I_{3}=\{3\}$. To add $ct_{add12}$ and $ct''$, the server expands them to $\bar{ct_{add12}}=\left( c_{0}+c_{0}^{'},c_{1},c_{1}^{'},0 \right)$ and $\bar{ct''}=(c_{0}^{''},0,0,c_{1}^{''})$. The server then outputs the ciphertext $ct_{add123}:=\bar{ct_{add12}}+\bar{ct''}$ = $\left( c_{0}+c_{0}^{'}+c_{0}^{''},c_{1},c_{1}^{'},c_{1}^{''} \right)\in R_{Q}^{4}$ and the combined index set $I_{1,2,3}=I_{1,2}\cup I_{3}=\{1,2,3\}$.

**Step 3.** Decryption Process

To decrypt $ct_{add123}:=\left( d_{0}.d_{1},d_{2},d_{3} \right)$, Parties 1, 2 and 3 must take part in the decryption step. For each $i\in\{1,2,3\}$, Party $i$ computes and broadcasts $d_{i}\cdot sk_{i}+e_{i}$ for some noise $e_{i}$. Then, each party can compute $d_{0}+\left( d_{1}\cdot sk_{1}+e_{1} \right)+\left( d_{2}\cdot sk_{2}+e_{2} \right)+\left( d_{3}\cdot sk_{3}+e_{3} \right)\approx\left( c_{0}+c_{1}\cdot sk_{1} \right)+\left( c_{0}^{'}+c_{1}^{'}\cdot sk_{2} \right)+\left( c_{0}^{''}+c_{1}^{''}\cdot sk_{3} \right)$, which approximates $\mu_{1}+\mu_{2}+\mu_{3}$.

**S.3. Multi-Key Homomorphic Multiplication**

To explain how multiplication is achieved in the Multi-Key CKKS scheme, we provide an illustrative example. Suppose we want to compute $m_{3}(m_{1}+m_{2})$, which is essentially equation S.3.1:

| $m_{3}\left( m_{1}+m_{2} \right)=\left( a_{3}+b_{3}s_{3} \right)\left( a_{1}+b_{1}s_{1}+a_{2}+b_{2}s_{2} \right)$  $= a_{3}\left( a_{1}+a_{2} \right)+a_{3}b_{1}s_{1}+a_{3}b_{2}s_{2}+b_{3}\left( a_{1}+a_{2} \right)s_{3}$  $+ b_{3}b_{1}s_{1}s_{3}+b_{3}b_{2}s_{3}s_{2}$ | S.3.1 |
| --- | --- |

Notice that we have quadratic terms: $s_{1}s_{3}$ and $s_{3}s_{2}$. If these quadratic terms can be linearized, $m_{3}(m_{1}+m_{2})$ will then be expressed as a linear combination of all secret keys, $s_{i}$ where $i=1 \sim3$. This would make ($A, B, C, D$) a valid encryption of $m_{3}(m_{1}+m_{2})$ as in equation S.3.2.

| $m_{3}\left( m_{1}+m_{2} \right)=(1, s_{1},s_{2},s_{3})\cdot(A,B,C,D)$ | S.3.2 |
| --- | --- |

Now, let us see how we can linearize $s_{1}s_{3}$, as an example. In essence, each party generating an additional set of secret & public key can linearize the quadratic terms. In our example of $s_{1}s_{3}$, Party 1 and Party 3 will do the following. Here, capitalized letters refer to public information:

1. Party 1 encrypts $s_{1}$ using $r_{1} \Longrightarrow s_{1}=Ar_{1}+D_{1}$
2. Party 1 encrypts $r_{1}$ using $s_{1} \Longrightarrow r_{1}=-U_{1}s_{1}-V_{1}$
3. Party 3 hides $s_{3} \Longrightarrow F_{3}=-As_{3}$

In the meantime, notice that $s_{1}s_{3}$ can be expressed as in equation S.3.3, since every term except $s_{1}s_{3}$cancels out:

| $s_{1}s_{3}=\left( -{As}_{3} \right)\left( -U_{1}s_{1}-r_{1} \right)+\left( -As_{3} \right)U_{1}s_{1}+\left( -{Ar}_{1}+s_{1} \right)s_{3}$ | S.3.3 |
| --- | --- |

Now, if you look carefully, equation S.3.3 is equivalent to equation S.3.4 after substituting, which is a linear combination of $s_{1}$ and $s_{3}$.

| $s_{1}s_{3}=F_{3}V_{1}+F_{3}U_{1}s_{1}+D_{1}s_{3}$ | S.3.4 |
| --- | --- |
|  |  |

**S.4. Derivation of Multiplicative Depth Minimization**

In this section, we demonstrate how equation (10) from the main manuscript achieves minimal multiplicative depth. To this end, we express the function $F$ that needs to be evaluated as shown in equation S.4.1, focusing on the highest order term, $\frac{\alpha}{n}x_{ij}c_{3}z_{i}^{3}$.

| $F=\frac{\alpha}{n}x_{ij} \left\{ c_{0}+c_{1}z_{i}+c_{3}z_{i}^{3}-y_{i} \right\}$ | S.4.1 |
| --- | --- |

If we compute the highest term naively, it will consume a total depth of **three**. This includes two from $c_{3}z_{i}^{3}$ and an additional one from the multiplication with $\frac{\alpha}{n}x_{ij}$. As detailed in equation S.4.2, the sequence of multiplications progresses from round to curly brackets, with the values within the same bracket type corresponding to the same multiplicative depth.

| $\left\{ \frac{\alpha}{n}x_{ij} \right\}\left\{ \left( c_{3}z_{i} \right)\left( z_{i}z_{i} \right) \right\}$ | S.4.2 |
| --- | --- |

However, by making the following observations, we can perform the same operation using only a depth of **two**, which is minimal depth consumption achievable given the third order term, $z_{i}^{3}$.

Firstly, we observe that it is strictly more efficient to combine the constants, $\frac{\alpha}{n}$ and $c_{3}$, into a single constant $\frac{\alpha}{n}c_{3}$. The server can encode $\frac{\alpha}{n}c_{3}$ without using any multiplicative depth, whereas encoding the two separately and then multiplying them would consume one depth. This results in $\left( \frac{\alpha}{n}c_{3} \right)x_{ij}z_{i}^{3}$, with the round bracket encapsulating this step of the process.

Secondly, we observe that obtaining $z_{i}^{3}$ from $z_{i}$ requires at least two depths. This can be achieved as outlined in equation S.4.3. Here, consistent with our earlier rule, the sequence of multiplications progresses from round to curly brackets to square brackets. The values within the same type of bracket correspond to the same multiplicative depth. Importantly, $z_{i}$ is already a product of the weight vector ($w$) and $x_{i}$, which means that $\left[ \left\{ \left( \frac{\alpha}{n}c_{3} \right)x_{ij} \right\}z_{i} \right]$ has the same multiplicative depth as $z_{i}^{2}$ in the next bracket. Ultimately, equation S.4.3 achieves a multiplicative depth consumption of two.

| $\left[ \left\{ \left( \frac{\alpha}{n}c_{3} \right)x_{ij} \right\}z_{i} \right]\cdot\left[ z_{i}^{2} \right]$ | S.4.3 |
| --- | --- |

We then formulate equation S.4.4 by adding the first order terms to equation S.4.3.

| $\left[ \left\{ \left( \frac{\alpha}{n}c_{3} \right)x_{ij} \right\}z_{i} \right]\cdot\left[ z_{i}^{2}+\frac{c_{1}}{c_{3}} \right]$ | S.4.4 |
| --- | --- |

Finally, we add the zeroth order terms to complete equation S.4.4. This results in equation S.4.5 that is equivalent to equation (1) in the main manuscript. Q.E.D.

| $\left[ \left\{ \left( \frac{\alpha}{n}c_{3} \right)x_{ij} \right\}z_{i} \right]\cdot\left[ z_{i}^{2}+\frac{c_{1}}{c_{3}} \right]+\left\{ \frac{\alpha}{n}\left( c_{0}-y_{i} \right) \right\}x_{ij}$ | S.4.5 |
| --- | --- |

**S.5. Background on Nesterov’s Accelerated Gradient Descent**

In the standard gradient descent algorithm, each iteration uses only the gradient value of the current point $w_{k}$. This can significantly slow the convergence speed as $w_{k}$ approaches the local minima. Nesterov proposed an improvement to address this issue ^3^. The Nesterov's accelerated gradient descent algorithm uses information from the previous point $w_{k-1}$ to build momentum in the update for the next point $w_{k+1}$. This results in a much faster convergence rate of $O(\frac{1}{k^{2}})$, compared to the $O(\frac{1}{k})$ rate of the regular gradient descent.

To further illustrate, consider the two equations below. equation S.5.1 is the intermediate update that looks ahead by approximating the next position of the parameter $w$. This is done using the current gradient information. Next, in equation S.5.2, the actual update is performed. This is achieved by a combination of the lookahead step $\beta_{k+1}$ and our previous lookahead step $\beta_{k}$. In this context, $\gamma_{k}$ acts as the momentum coefficient, controlling the influence of the previous update on the current one.

| $\beta_{k+1}=w_{k}-\frac{\alpha}{n}\frac{\partial NLL\left( w_{k} \right)}{\partial w_{k}}$ | S.5.1 |
| --- | --- |

| $w_{k+1}=\left( 1-\gamma_{k} \right)\beta_{k+1}+\gamma_{k}\beta_{k}$ | S.5.2 |
| --- | --- |

**Bibliography**

1 Regev, O. On lattices, learning with errors, random linear codes, and cryptography. *Journal of the ACM (JACM)* **56**, 1-40 (2009).

2 Brakerski, Z., Langlois, A., Peikert, C., Regev, O. & Stehlé, D. in *Proceedings of the forty-fifth annual ACM symposium on Theory of computing.* 575-584.

3 Nesterov, Y. A method of solving a convex programming problem with convergence rate O (1/k** 2). *Doklady Akademii Nauk SSSR* **269**, 543 (1983).
